# First Serological Evidence of Crimean-Congo Hemorrhagic Fever Virus and Rift Valley Fever Virus in Ruminants in Tunisia

**DOI:** 10.3390/pathogens10060769

**Published:** 2021-06-18

**Authors:** Khaoula Zouaghi, Ali Bouattour, Hajer Aounallah, Rebecca Surtees, Eva Krause, Janine Michel, Aymen Mamlouk, Andreas Nitsche, Youmna M’ghirbi

**Affiliations:** 1 Laboratory of Viruses Vectors and Hosts (LR20IPT02), Institute Pasteur of Tunis, University of Tunis El Manar, Tunis 1002, Tunisia; khawlazouaghi@gmail.com (K.Z.); ali.bouattour@pasteur.tn (A.B.); aounallahhajer@gmail.com (H.A.); 2 Division Highly Pathogenic Viruses, Centre for Biological Threats and Special Pathogens, Robert Koch Institute, Seestraße 10, 13353 Berlin, Germany; SurteesR@rki.de (R.S.); KrauseE@rki.de (E.K.); MichelJ@rki.de (J.M.); NitscheA@rki.de (A.N.); 3 Service de Microbiologie et Immunologie, Ecole Nationale de Médecine Vétérinaire, Université de Manouba, Sidi Thabet 2020, Tunisia; aymen.mamlouk@enmv.uma.tn

**Keywords:** Crimean-Congo hemorrhagic fever virus, Rift Valley fever virus, enzyme-linked immunosorbent assays, indirect immunofluorescence assay, risk factors, ruminants, Tunisia

## Abstract

Crimean-Congo hemorrhagic fever virus (CCHFV, *Nairoviridae* family) and Rift Valley fever virus (RVFV, *Phenuiviridae* family) are zoonotic vector-borne pathogens with clinical relevance worldwide. Our study aimed to determine seroprevalences of these viruses and potential risk factors among livestock (cattle, sheep, and goats) in Tunisia. Sera were tested for antibodies against CCHFV (n = 879) and RVFV (n = 699) using various enzyme-linked immunosorbent assays (ELISAs) and indirect immunofluorescence assays (IIFA). The overall seroprevalence of IgG antibodies was 8.6% (76/879) and 2.3% (16/699) against CCHFV and RVFV, respectively. For CCHF seropositivity bioclimatic zones and breed were potential risk factors for the three tested animal species; while the season was associated with cattle and sheep seropositivity, tick infestation was associated with cattle and goats seropositivity and age as a risk factor was only associated with cattle seropositivity. Age and season were significantly associated with RVFV seropositivity in sheep. Our results confirm the circulation of CCHFV and RVFV in Tunisia and identified the principal risk factors in ruminants. This knowledge could help to mitigate the risk of ruminant infections and subsequently also human infections.

## 1. Introduction

Crimean-Congo hemorrhagic fever virus (CCHFV, *Nairoviridae* family) and Rift Valley Fever virus (RVFV, *Phenuiviridae* family) are the causative agents of Crimean-Congo hemorrhagic fever (CCHF) and Rift Valley fever (RVF), respectively. These viruses belonging to the *Bunyavirales* order have clinical relevance worldwide [1].

CCHFV of the *Orthonavirus* genus is transmitted to large and small mammals and birds(with the exception of ostriches), by ticks [2]. Humans are infected by tick bites, or through exposure to blood or infected tissues from viremic animals, or infected persons [3]. Only 12% of infected people will develop symptoms. Acute CCHF in humans is characterized by an onset of fever, tremors, myalgia, headaches, nausea and vomiting, abdominal pain and arthralgia. In severe cases, the disease is complicated by the appearance of bleeding from mucosal membranes (e.g., nose and vagina) and ecchymosis; lethality rates can reach 9–50% [4,5]. In contrast, CCHFV infection in wild and domestic mammals does not cause severe disease, in general, infected animals remain asymptomatic [6]; but they play an essential role in the amplification and spread of the virus [7,8]. Among these animals, domestic ruminant species are considered to be an important indicator of CCHFV circulation in a naïve area [9]. The geographic distribution of CCHFV coincides with that of ixodid ticks; mainly those of the genus *Hyalomma*, the principal vectors of CCHFV. Indeed, CCHF has been reported in more than 30 countries in Africa, Asia, and the Middle East, and has shown a significant increase in prevalence during the last few decades in Eastern Europe [3,10]. However, CCHFV might expand outside its current geographic range through the introduction of infected ticks by migratory birds into naïve regions, or through the international livestock trade. Moreover, increasing international travel to CCHFV endemic areas, climate change, and increasing human population densities may escalate the worldwide spread of CCHFV [11]. Recently, there has been a worrying emergence of CCHF in Spain, with six human cases (three fatal) reported in rural areas since 2016, the last one in August 2020 [12] due to viral transmission from an infected *Hyalomma* tick. In North Africa, CCHFV nucleic acid has been detected in ticks infesting migratory birds in Morocco [13] and in *Hyalomma aegyptium* ticks collected from tortoises in Algeria [14]. In addition, anti-CCHFV antibodies were detected in serum samples collected from camels, domestic cattle, buffalo, sheep and goats from Egypt [15,16]. In Tunisia, a survey was conducted in 2014 in febrile patients with acute fever (n = 181), only five of whom showed anti-CCHFV IgM antibodies with no history of foreign travel to known CCHFV endemic areas, nor a history of tick bites. Additionally anti-CCHFV IgG antibodies were detected in two out of thirty-eight tested slaughterhouse workers [17]. More recently, a highly unexpected seroprevalence of CCHFV (89.7%) was reported in camels (*Camelus dromedarius*) in Southern Tunisia [18]. These results paved the way for further investigations into this highly pathogenic virus in Tunisia, especially with the presence of potential vectors of CCHFV, *Hyalomma* spp., in the Mediterranean basin, and the absence of routine animal surveillance in Tunisia.

Rift Valley fever virus is a mosquito-borne virus of the *Phlebovirus* genus that affects not only cattle, sheep, goats and camels, but also humans and wildlife [19]. Unlike CCHFV, RVFV is highly pathogenic in ruminants and regularly causes devastating abortion storms, and deaths of young animals, resulting in subsequent economic losses of livestock during an outbreak [20]. In addition, fetal death resulting from vertical transmission of the virus has been documented in humans [21,22].

Transmission of this virus to humans occurs by contact with blood, body fluids, or tissues of infected animals, or through the bite of infected mosquitoes [20]. This virus has been isolated from more than 50 mosquito species belonging to seven different genera; however, the principal vectors for RVFV are *Culex* spp. and *Aedes* spp. [23,24]. Rift Valley fever disease in humans is typified by a benign febrile illness, but some patients may develop fatal hemorrhagic fever, encephalitis, or ocular sequelae [25].

RVFV is an emerging zoonotic threat to veterinary and public health with the potential to have an important socioeconomic impact. RVF disease has expanded its historic geographic range in the livestock-raising areas of eastern and southern Africa and into the Middle East (Saudi Arabia and Yemen) over the past 25 years, causing several epizootics and epidemics. The first reported case was in 1931 in Kenya [26]; subsequent epizootics/epidemics were reported in South Africa in 1951 [27] and in other Sub-Saharan countries in Africa such as Zimbabwe in 1970 [20] and Sudan in 2007 [28]. The virus range has now expanded to include North Africa where the largest epidemic occurred in Egypt in 1977–1978 [29], and four large outbreaks of RVF occurred in Mauritania in 1998, 2003, 2010 and 2012, which resulted in a high number of animal and several human deaths. In Tunisia, a study carried out between 2006 and 2007 did not reveal any exposure of ruminants to RVFV [30]. However, recently it has been shown that among 470 serum samples of apparently healthy camels in Tunisia, 162 were RVFV seropositive (34%) [31]. In addition, in the summer of 2014 active circulation of RVFV was reported in humans in Tunisia, suggesting their evident exposure to this virus in Tunisia [32]. In North Africa, the vector competence of different mosquito species, such as *Culex pipiens*, to transmit RVFV has been established [33,34,35].

Given the many uncontrolled border crossings that take place between Tunisia and its neighboring countries, Libya and Algeria, livestock exchanges may happen with countries where CCHFV and RVFV are endemic and where no active surveillance is practiced by local sanitary services. Social and political instability in these neighboring countries, can favor the accidental transportation of animals infected with CCHFV and RVFV into Tunisia, where known vectors of these viruses, ticks of the *Hyalomma* genus and mosquitoes of the *Culex* and *Aedes* genus, are endemic [36,37]. Therefore, Tunisia can be considered to be a country at high risk for the establishment of CCHFV and RVFV [38]. To our knowledge no study has been carried out to investigate the presence or the absence of these two viruses in cattle, sheep and goats in Tunisia. The detection of specific antibodies against these two viruses in these animal species in Tunisia constitutes a good indicator for their presence. Thus, we aimed to investigate the evidence of circulation of CCHFV and RVFV in ruminants (cattle, sheep and goats) in different bioclimatic zones in Tunisia, in order to estimate their seroprevalence, and to identify the potential risk factors associated with these arboviruses.

## 2. Results

### 2.1. Serological Investigation

Serum samples collected from cattle, sheep and goats in various bioclimatic zones (humid, semi-humid, semi-arid and arid) across North and Central Tunisia were tested by enzyme-linked immunosorbent assays (ELISAs) for antibodies targeting CCHFV (n = 879) or RVFV (n = 699). Details regarding the animal and geographic origin of the serum samples are given in Table 1. All tested animals were apparently healthy, and the majority were born and reared in Tunisia, however according to the circumstances (e.g., abundant pasture) the farmers introduce imported animals into their herds for fattening. Serum samples that tested positive or were inconclusive in the ELISAs were further assayed using a CCHFV IgG indirect immunofluorescence assay (IIFA) or RVFV IgG IIFA. The results of the IIFA were considered the final results for the presence or the absence of CCHFV or RVFV specific IgG antibodies. In addition, serum samples that tested positive in the RVFV competitive ELISA (cELISA) were then screened using a RVFV IgM capture ELISA.

Among 97 sera detected positive by CCHFV ELISA, only 76 samples were confirmed positive by IIFA. In contrast, all inconclusive ELISA samples (n = 6) tested negative by IIFA. For anti-RVFV antibody detection, among 20 cELISA seropositive and 10 inconclusive sera, only 14 and 2 samples respectively, were confirmed seropositive using IIFA.

The overall seroprevalence of CCHFV antibodies was 8.6% [95% CI: 6.78–10.5%]. The seroprevalence ranged from 0% (n = 0/271) [95% CI: 0–1.1%] to 23.4% (n = 51/218) [95% CI: 17.77–29.01%] depending on the bioclimatic zone (Table 2). CCHFV seroprevalence varied by species, with cattle at 11.1% (43/388) [95% CI: 7.95–14.2%], sheep 6.2 % (20/325) [95% CI: 3.54–8.76%] and goats 7.8% (13/166) [95% CI: 3.74–11.91%] (Table 2).

For RVFV antibodies, 3.6% [95% CI: 0.51–6.79%] of tested ruminants were seropositive in sub-humid zone, 2.8% [95% CI: 1.01–4.66%] in the semi-arid zone, 0.9% [95% CI: 0–2.15%] in the humid zone and 0% [95% CI: 0–12.5%] in the arid zone (Table 2). A total of 16 serum samples including 10 cattle (3.3%; [95% CI: 1.3–5.38%]) and 6 sheep (2.6%; [95% CI: 0.53–4.57%]) were seropositive, while no RVFV-specific antibodies were detected in goats (Table 2). The screening of these 16 positive sera using the ID screen Rift Valley fever IgM capture ELISA (Innovative Diagnostics, France) revealed the presence of IgM antibodies in only one bovine serum originating from Oued Abid located in the sub humid zone. No serum samples were positive for both anti-CCHFV and anti-RVFV antibodies.

### 2.2. Risk Factor Analysis

In order to identify risk factors associated with CCHFV and RVFV seropositivity in Tunisian ruminants, information such as animal breed, age, sex, season, bioclimatic zone, tick infestation and type of breeding (cattle only) was acquired, and statistically analyzed together with the seroprevalence rates. The univariate logistic analysis showed that cattle breed (*p*-value = 0.005), age (*p*-value = 0.007; OR = 0.259; [CI%: 0.09–0.743]), season (*p*-value = 0.001), bioclimatic zone (*p*-value < 0.001), and tick infestation (*p*-value < 0.001; OR = 17.489; [CI%: 4.929–62.051]), were significantly associated with CCHF seropositivity in cattle (Table 3). In contrast, the sex (*p*-value = 0.087) was not a significant risk factor for CCHFV seropositivity among cattle (Table 3). Among 325 sheep tested for CCHFV-specific antibodies, only 20 were seropositive (Table 3) with a significant association with breed (*p*-value < 0.001), season (*p*-value = 0.008) and bioclimatic zone (*p*-value < 0.001) (Table 3). The data illustrated that age (*p*-value = 0.476) and sex (*p*-value = 0.784), were not significant risk factors for CCHFV seropositivity among sheep (Table 3).

The results of the statistical analysis in Table 3 show a significant association between goats’ breed (*p*-value = 0.023), bioclimatic zone (*p*-value < 0.001), tick infestation (*p*-value < 0.001; OR = 35; [CI%: 7.173–170.773]) and CCHFV seropositivity. The remaining tested risk factors such as age, sex and season did not show a significant association with seropositivity (*p*-value > 0.05) (Table 3).

In contrast, there was no significant association between any of the different risk factors in cattle and RVFV seropositivity (Table 4). Among the six RVFV seropositive sheep, only age and season (*p*-value < 0.001), were significantly associated with seropositivity (Table 4).

## 3. Discussion

In this study, the serological screening of animals revealed for the first time in Tunisia, the presence of anti-CCHFV and anti-RVFV antibodies in cattle, sheep, and goats, with an overall seroprevalence of 8.6% (76/879) and 2.3% (16/699), respectively. To our knowledge, only one previous study has reported CCHFV-specific antibodies in humans in Tunisia. This survey was conducted in 2014 and reported the presence of anti-CCHFV IgM titers in febrile patients (n = 5) with acute fever, and no history of foreign travel to known CCHFV endemic areas, nor a history of tick bites. In addition, anti-CCHFV IgG antibodies were detected in two out of thirty-eight tested slaughterhouse workers [17]. More recently, an unexpectedly high seroprevalence of CCHFV was reported in camels in Southern Tunisia [18]. In addition, these camels were reported to be infested by CCHFV infected *Hyalomma impeltatum* ticks [18]. Our results confirm these previous studies and suggest that CCHFV is circulating in Tunisia, albeit at a very low level. In fact, CCHFV was also detected in neighboring countries, in *Hyalomma aegyptium* ticks collected from tortoises in Algeria [14], and in *Hyalomma marginatum* ticks collected from birds in Morocco [13]. This finding is not surprising since a recent model generated by Okely et al. (2020) [38] predicted a high environmental suitability for CCHFV occurrence in northwestern Africa, including Tunisia, Algeria, and Morocco. However, to date, no human clinical cases of CCHF have been observed in North Africa. This could be explained by the lack of diagnostic testing in humans, or it could be that the CCHFV strain circulating in our region is not pathogenic for humans. Indeed, Kautman et al. (2016) [14] reported the circulation of a non-pathogenic CCHFV strain AP92 (lineage Europe 2) in *H. aegyptium* collected from tortoises in Algeria, which is associated with sub-clinical or mild cases [39,40]. The AP92 strain, described for the first time in *Rhipicephalus bursa* ticks in Greece in 1975 [41], was also detected in Turkey [42] and Kosovo [43] causing asymptomatic infections. On the other hand, the detection of CCHFV and subsequent clinical cases could also be expected in Tunisia, since a similar epidemiological situation was observed in Spain until the first CCHFV outbreak in 2016 [44,45].

Our results revealed different CCHFV seroprevalence rates between cattle (11.1%; 43/388), and small ruminants (6.2% sheep, 7.8% goats). This result can be explained by the fact that *Hyalomma* species, the known common vector of CCHFV, is one of the major tick species infesting cattle in Tunisia [36]. In addition, cattle tend to be highly infested with *Hyalomma* spp., about ten times higher than small ruminants [46]. Similar CCHFV seroprevalence results were also reported in Sudan (19.14% of cattle tested were seropositive [47]), Bulgaria and Greece (6% cattle; 1% sheep were seropositive [48]) and France Corse (13.3% cattle; goats 3.1% and 2.5% sheep) [49], indicating that cattle can be a good animal species indicator for seroepidemiological CCHFV monitoring studies. In contrast, our findings differ from the results of Schuster et al. (2016) [50] which highlighted the suitability of small ruminants as an indicator for the presence of CCHFV.

In our study, we investigated different risk factors (breed, age, sex, season, bioclimatic zone, type of breeding, and tick infestation) associated with CCHFV seropositivity. Only two factors (bioclimatic zone and breed) showed a positive correlation with seropositivity in all tested animal species (*p*-value < 0.05).

The overall seroprevalence for CCHFV in the three analyzed animal species ranged from 0% to 29.4%, depending on the bioclimatic zone, with the highest seroprevalence rate in the sub-humid zone. However, this result is not surprising since bioclimatic factors affect the distribution of tick vectors, and therefore the occurrence of the virus [51,52]. The higher CCHFV seroprevalence in the sub-humid zone could be explained by the abundance of ticks of the *Hyalomma* species in this bioclimatic zone. Indeed, a positive correlation was found between the tick infestation of the tested ruminants and the rate of CCHFV seropositivity (*p*-value < 0.001). This result was not surprising given that livestock animals serve as a host for tick populations, among them vectors of CCHFV. Moreover, CCHFV has been detected in numerous tick genera, including *Hyalomma*, *Rhipicephalus*, *Boophilus*, *Dermacentor*, *Ambylomma*, and *Haemaphysalis* [9,53]. In Tunisia, among the 14 species of the genera *Hyalomma*, *Rhipicephalus*, *Dermacentor*, *Haemaphysalis* and *Ixodes* that infest livestock, *H. marginatum* and *H. excavatum* are the most abundant and widespread in all bioclimatic zones [36,54]. In fact, CCHFV is mostly transmitted by *Hyalomma* ticks in several endemic countries such as Greece [55], Turkey [56,57], Albania [58], Iran [59], and Kosovo [60], as well as in countries neighboring Tunisia, such as Algeria [14] and Morocco [13].

In addition, our survey revealed a significant association between breed and CCHFV seroprevalence. In cattle, the highest seroprevalence was observed among local breeds (21.6%). This result might be attributed to differences in their management system, local breeds were left grazing in the field all day, whereas the other analyzed breeds were kept indoors with a good management system for dairy production. Hence, they were at lower risk of disease. This finding is in line with reports from Ghana and Ethiopia [61,62]. In contrast, the- situation is different in Sudan where the highest CCHFV seroprevalence rate was observed among cross breeds [63]. In sheep, we found a high seroprevalence of CCHFV in the Queue Fine de l’Ouest (QFO) breed (56%). This result can be explained by the high tick infestation rate of the QFO breed compared to the other tested breeds (Barbarine, Black Tibar and cross breeds). This agrees with the finding of Elati et al., (2018) [64] who report that the QFO sheep breed were more infested by ticks in Tunisia than other breeds. However, breed-specific epidemiological patterns of tick infestation and CCHFV infection need deeper investigation.

A significant correlation between CCHFV seropositivity and the season was observed in cattle (*p*-value = 0.001) and sheep (*p*-value = 0.008). The observed seroprevalence is higher in summer and autumn compared to spring and winter, corresponding to the seasons of transmission for tick borne pathogens. This is mainly related to the bioclimatic needs of *Hyalomma* spp. life cycle in Tunisia, making this tick genus more abundant on hosts in the summer and autumn compared to the spring and winter [35]. However, in our study we could not confirm the detection of anti-CCHFV IgM antibodies (which would indicate a recent infection with CCHFV), therefore further extensive studies are needed to confirm the seroprevalences of both anti-CCHFV IgM and IgG antibodies in order to investigate this potential seasonal variation in seropositivity. Previous studies have reported that outdoor grazing for livestock during summer months increases the opportunity for them to become infested by ticks [65,66].

According to our results, CCHFV seroprevalence increases with cattle age. This can be explained by the cumulative exposure of older cattle to tick infestation since they graze in an open system [47,63,67]. Similar results were observed in Sudan, Egypt, Iran, and Ethiopia, demonstrating that there is generally a lower risk of young calves becoming infected with CCHFV [16,62,63,68]. Besides, CCHFV IgG antibodies persist for a long time, which supports seropositivity being significantly associated with age [69]. There was no significant association between sex and CCHFV seroprevalence. A similar observation was also reported in Sudan [63].

In our study, we provide the first seroepidemiological data on RVFV circulation in cattle, sheep and goats in Tunisia. We report the presence of specific IgG and IgM antibodies against RVFV in cattle and sheep in Tunisia, contrary to the findings of Ayari Fakhfekh et al. (2011) [30], who did not detected antibodies against RVFV in tested small ruminants between September 2006 and January 2007 in Tunisia. However, a recent study conducted on camels in Tunisia between January 2017 and December 2018 indicated that 34% of tested animals have IgG antibodies against RVFV [31]. In addition, a serosurvey of RVFV conducted in Tunisia on febrile patients, non-febrile healthy agriculture workers, and slaughterhouse workers during summer 2014 revealed evidence of human exposure to RVFV [32]. These findings may imply an active circulation of RVFV in Tunisia. This is not surprising since previous studies have reported a seroprevalence of 0.97% and 15% among camels in the neighboring countries Algeria and Morocco, respectively [70,71]. The introduction of RVFV into Tunisia from these neighboring countries may occur during an outbreak, due to the uncontrolled and illegal transportation of livestock in the border regions, as well as the risk posed by the introduction of infected mosquitoes. This illegal livestock transportation can also enhance the transmission and spread of other transboundary animal diseases, such as foot and mouth disease. Therefore, it is likely that the abundant trading in ruminants between Tunisia and neighboring countries (Algeria, Libya) may explain the seropositive results in this study. Indeed, Saudi Arabia’s outbreak in 2000 was due to the uncontrolled introduction of infected ruminants from eastern Africa during the Hajj [72]. Other studies have suggested that formal and informal exchanges of domestic animals in border regions increase the spread of this disease, as is the case between Mauritania and southern Algeria, and Ethiopia and Southern Sudan [73,74]. Supporting our hypothesis, previous studies evaluating the spatial and temporal suitability of regions in North Africa for sustaining RVFV circulation, showed that the northern regions of the Maghreb are moderately suitable for RVFV enzootics and highly suitable for RVFV epizootics [75,76]. These risk areas extend along the coasts and into the Atlas Mountains in Morocco, Algeria, and Tunisia [75,76]. The suitability of these countries to support the circulation of RVFV can be attributed to the abundance and widespread presence of its most efficient mosquito vector, *Culex pipiens* [35,37,77]. Moreover, Amraoui et al. (2012) [35] confirmed experimentally that *Culex pipiens* populations collected in Algeria, Morocco, and Tunisia, were highly suitable for the transmission of RVFV [35]. In addition, several characteristics of *Aedes* and *Culex* mosquitoes are particularly relevant showing their RVFV dissemination capacity [33,78]. The presence of *A. albopictus* [79] and the competent RVFV vector *Cx. pipiens* [32] in Tunisia increase the potential risk for RVFV establishment. The introduction of RVFV into Mediterranean basin countries potentially makes this virus an extensive public health burden.

Our study showed a correlation between age and RVFV seropositivity in sheep, with young animals (six months to one year) having an increased probability of being seropositive compared to older animals. This is not surprising as sheep start grazing with adults from six to seven months, and are therefore more exposed to mosquitos and the viruses they transmit at this age. Our results agree with a previously reported study in northern Somalia, suggesting that young sheep are more susceptible to RVFV infection [80]. In contrast, other studies have shown that RVFV seroprevalence is higher in adult animals [81,82,83].

A significant correlation between RVFV IgG seroprevalence and season (*p*-value = 0.05) was also observed in sheep. However, the seroprevalence reported in autumn is expected as it coincides with the abundance of the vector *Cx. pipiens* [37]. This vector abundance has been associated with the favorable weather and increased precipitation, which creates larval breeding habitats for mosquitoes. IgM antibodies against RVFV were detected in only one bovine serum sample originating from Oued Abid located in the sub humid zone. This result indicates that there was at least one recent RVFV infection during the sampling period. In general, RVFV-IgM antibodies can persist in the host only 14 days post-infection [84]. However, other studies suggest that IgM antibodies can be detected for a maximum duration period of two months after infection [85,86]. Given that our study revealed a total of sixteen serum samples including ten cattle (3.3%; [95% CI: 1.3–5.38%]) and six sheep (2.6%; [95% CI: 0.53–4.57%]) to be seropositive, it is not unexpected that one of these serum samples was IgM as well as IgG positive, as these results imply a low-level circulation of RVFV in ruminants in Tunisia.

Serological tests such as ELISAs are often based on recombinant nucleocapsid proteins (NP) and offer high specificity, simple sample processing and are relatively high throughput as they typically use a 96-well plate format. Indirect immunofluorescent tests (IIFTs), though typically less high throughput, have the added advantage that additional information regarding the specificity of the antibodies can be gained by analysing the fluorescence distribution patterns in the infected/transfected cells. However, serum neutralization tests (NT) are typically considered to be the “gold standard” in viral serological assays as they are highly specific and provide information regarding the functionality of the antibodies in the serum sample. One on the limitations of this study was the inability to confirm both CCHFV and RVFV seropositive samples by serum neutralization test. It is possible that we could have reported false positive results in our study, however, the use of both ELISA and IIFT to confirm the presence of anti-RVFV and anti-CCHFV IgG antibodies greatly reduces the likelihood of this happening. The lack of a variety of serological kits to test for antibodies against CCHFV and RVFV in animal serum samples necessitated the adaption of IIFT kits designed to detect anti-CCHFV and anti-RVFV antibodies from human serum samples, in order to confirm the samples that tested positive by ELISA in this study. The IIFT kits were adapted according to previously published protocols to detect ruminant IgG antibodies [50,87,88], however the sensitivity and specificity of the adapted kits are unknown. Unfortunately, we could not adapt the IIFT kit to detect anti-RVFV IgM antibodies to confirm the sample that tested positive for anti-RVFV IgM antibodies by ELISA. Another limitation of this study was the inability to detect anti-CCHFV IgM antibodies, which would add valuable evidence for the circulation of this virus in ruminants in Tunisia. The retrospective nature of the study also limited the additional data that could be used for the univariate risk analysis.

These results lay the foundations for further studies which would benefit from testing more serum samples for the presence of both IgG and IgM antibodies against CCHFV and RVFV, as well as the use of an RT-qPCR assay to detect CCHFV and RVFV RNA genomes, which would provide direct evidence of the circulation of these viruses in Tunisia.

## 4. Materials and Methods

### 4.1. Experimental Design

**Figure 1:**
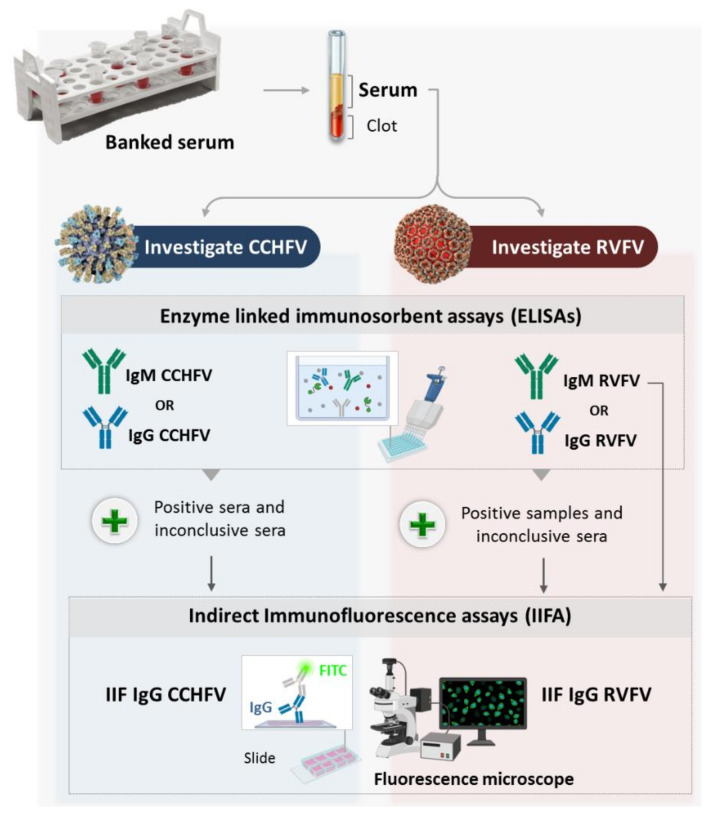
Schematic representation of the experimental design.

We used enzyme-linked immunosorbent assays (ELISAs) and indirect immunofluorescence assays (IIFA) to test for CCHFV and RVFV specific antibodies in sera collected from cattle, sheep and goats in Tunisia. The used workflow is summarized in Figure 1.

### 4.2. Study Area

**Figure 2:**
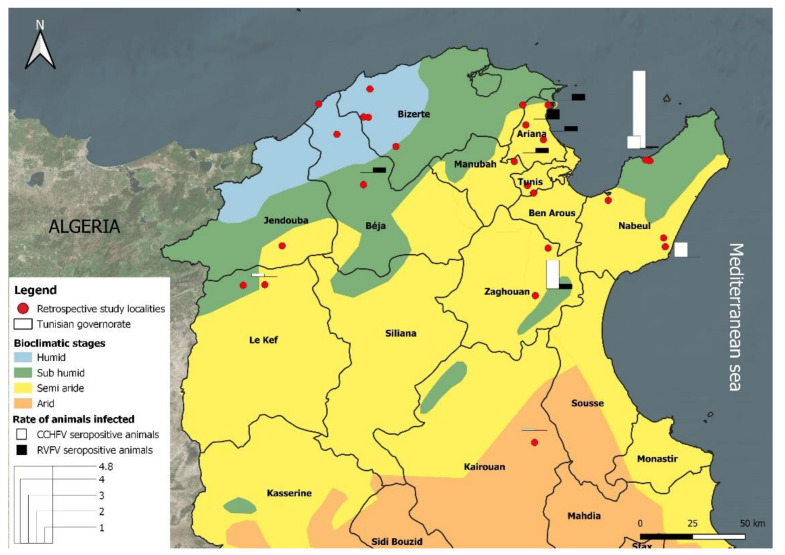
Bioclimatic map of Tunisia showing localities of sera collection.

A retrospective serosurvey in cattle, sheep and goats was carried out using banked sera samples that were collected between 2011 and 2014 in the frame of the RESTUS project (Project n°2AS1.3/023)aiming to strengthen and improve the surveillance of emerging zoonotic vector-borne diseases caused by bacteria (*Anaplasma phagocytophilum*, *Bartonella* spp., *Coxiella burnetii* and *Rickettsia* spp.) and viruses (West Nile virus and Rift Valley Fever virus).The tested banked sera were selected by simple random sampling. These serum samples were collected from 26 localities belonging to four bioclimatic zones (humid, sub-humid, semi-arid and arid) located in North and Central Tunisia (Figure 2 and Table 1).

The humid zone is characterized by an annual rainfall of between 800 and 1200 mm, while the sub-humid zone’s rainfall ranges between 500 and 700 mm per year. In these zones, located in the north of the country, rainfall is significantly higher than in the semi-arid and arid zones. The humid zone is mountainous with natural vegetation formed mainly by cork forests (*Quercus suber*, *Q. faginea*) while the sub-humid zone’s natural vegetation is mainly *Olea europea*, *Pistacia lentiscus*, *Q. suber* and *Ceratonia siliqua*, associated with annual crops (wheat), grazing areas and rangeland. More than 65% of the cattle herds are found in these two zones, where the tick fauna is varied and composed of species belonging to five genera (*Hyalomma*, *Rhipicephalus*, *Ixodes*, *Dermacentor* and *Haemaphysalis*) [36].

In the semi-arid zone, rainfall ranges between 200 and 500 mm per year, while in the arid zone rainfall is less than 200 mm per year. In these two bioclimatic zones, situated in northern and southern parts of the country, temperatures are generally high, and land is degraded due to deforestation and desertification. In the highlands the main native plant species are *Quercus ilex*, *Pinus halepensis* and *Juniperus phoenicea* [36]. In the arid (lowlands), the vegetation is dominated by *Stipa tenacissima, S. parviflora,* and *Artemisia herba* [36]. About 60% of the sheep and goats are grazed in the center of the country (semi-arid and arid areas) with traditional system (ranching) of production. Sheep and goats traditionally graze on hillsides and steppes in winter and stubble in summer. Tick fauna is limited to a few species belonging to the *Hyalomma* and *Rhipicephalus* genera.

Details relating to the animals’ breed, age, sex, season, bioclimatic zone, type of breeding and tick infestation status were obtained from the RESTUS project databases. All studied animals were apparently healthy, and the majority were born and reared in Tunisia, however according to the circumstances (e.g., abundant pasture) the farmers introduce imported animals into their herds for fattening. Tunisia’s climate is a Mediterranean climate with cool, moist winters and dry, hot summers. Rainfall and temperatures vary considerably from north to south; the latter is bordered by the Sahara Desert. Consequently, there is a declining rainfall gradient from north to south.

### 4.3. Serum Samples

Investigated banked sera were selected by simple random sampling to be screened for antibodies against CCHFV and RVFV. A total of 879 serum samples from cattle (n = 328), sheep (n = 325), and goats (n = 166), were tested for CCHFV antibodies and 699 among them were screened against RVFV antibodies (cattle n = 299, sheep n = 235, and goats n = 165) (Table 1). The age of the cattle ranged between 1 to 13 years and was classified into two categories: young (≤18 months) and adults (>18 months) while small ruminants were classified as either young (6 months to 1 year in order to exclude animals with maternal antibodies) or adults (>1 year) (Table 3 and Table 4).

### 4.4. Serological Investigation

#### 4.4.1. CCHFV and RVFV Enzyme Linked Immunosorbent Assays (ELISAs)

Serum samples were tested using the commercial ID Screen^®^ Rift Valley Fever Competition Multispecies ELISA kit (cELISA; Innovative Diagnostics; Montpellier, France) for the detection of RVFV antibodies (IgM/IgG) and ID Screen^®^ CCHF Double Antigen Multi-species ELISA (Innovative Diagnostics; Montpellier, France) for the detection of CCHFV antibodies (IgM/IgG). According to previous assays, both kits have a validated diagnostic sensitivity and specificity [89,90]. The diagnostic sensitivity and specificity of the ID Screen^®^ RVFV cELISA was found to be 98% and 100%, respectively, by most labs which participated in a European ring trial evaluating ELISAs used for RVFV diagnosis. The diagnostic sensitivity of the ID Screen^®^ CCHF Double Antigen Multi-species ELISA is reported to be 98.9% (CI (95%): 96.8–99.8%) and the diagnostic specificity 100% (CI (95%): 99.8–100%) [89,90]. The target antigen in both ELISAs is the nucleocapsid protein of the respective virus. ELISA protocols were conducted according to manufacturer’s instructions and sera were tested in pools of two serum samples. The OD for each sample was read at a wavelength of 450 nm. The results of the anti-CCHFV and anti-RVFV antibody detection were calculated as a percentage of the positive or negative control, respectively, as indicated by the manufacturer.

#### 4.4.2. CCHFV and RVFV Indirect Immunofluorescence Assays (IIFA)

Based on the performance of the IgG/IgM ELISAs, sera with a negative result were interpreted as negative. All sera with positive or inconclusive results using IgG/IgM ELISA kits were analyzed by confirmatory modified commercial IgG CCHFV and RVFV IIFA kits (Euroimmun, Lübeck, Germany) according to the previously published protocol [50,87,88]. Pooled serum samples were tested individually by IIFA. Briefly, sera were diluted 1/20 and 1/100, respectively in sample buffer (ready for use). A total of 25 µL of diluted sera was incubated with the IIFA slides, which contained either a mixture of RVFV infected and non-infected Vero E6 cells (in the case of the RVFV IIFA), or CCHFV-GPC or CCHFV-NP transfected and non-transfected cells (in the case of the CCHFV IIFA) for 30 min at room temperature. All washing steps (10 min each) were performed in PBS-Tween 20. After the first wash, 25 µL of rabbit anti-bovine IgG Fluorescein isothiocyanate (FITC) conjugate (Sigma-Aldrich, France) or rabbit anti-goat IgG FITC conjugate (Sigma-Aldrich, France) or rabbit anti-sheep IgG FITC conjugate (Sigma-Aldrich, France) (used for cattle, goat and sheep sera, respectively) diluted 1/200 in PBS-Tween 20 (containing 0.005% Evans blue) was added to each slide. Following a second washing step, the slides were dried, and glycerin was added. The slides were visualized with a fluorescence microscope (Leica microscope dm 1000 led, Wetzlar, Germany).

#### 4.4.3. RVFV-IgM ELISA

Positive samples identified using the RVFV cELISA kit were then tested by the ID screen^®^ Rift Valley Fever IgM capture ELISA kit (Innovative Diagnostics; Montpellier, France) following the manufacturer’s instructions, in order to detect RVFV IgM antibodies.

IgM antibodies directed against the nucleocapsid protein (NP) of CCHFV were not tested due to the absence of a valid commercialized kit.

### 4.5. Statistical Analysis

Descriptive epidemiological measures were calculated using statistical software IBM SPSS (Version 23.0. IBM Corp: Armonk, NY, USA) to determine the seroprevalence of CCHFV and RVFV in the different study sites with 95% confidence intervals (CI). Univariate analysis was performed and the association between seropositivity and risk factors (breed, age, sex, season, bioclimatic zone, type of breeding, and tick infestation) was assessed using the chi-square test. Statistical significance was defined as a two-tailed *p*-value < 0.05.

## 5. Conclusions

Our study confirms previous CCHFV and RVFV infection among native ruminants in Tunisia. These results support the implementation of an early detection system and the active control of these diseases in Tunisia to protect human population. In the case of RVFV, vaccination could constitute a solution to protect livestock, since both inactivated and live-attenuated vaccines have been approved for veterinary use. The reported seroprevalences raise an important public health threat that needs to be further explored by conducting fine-scale eco-epidemiological studies to investigate the dynamics of the vector-host-environment interaction of these viruses in Tunisia and its neighboring countries Algeria and Libya. This will allow us to better understand the risk factors for infection in an ecosystem that has a high probability of becoming endemic for these viruses. Besides, the high-risk human groups (e.g., slaughterhouse workers and butchers) and the *Hyalomma* tick distribution should be considered to better predict and respond to eventual CCHF and RVF human cases.

## Figures and Tables

**Table 1:** Number of tested samples for CCHFV and RVFV antibody detection.

Bioclimatic Zones	Governorates	Localities	Number of Tested Animals [CCHFV] (RVFV)			
			Cattle	Sheep	Goats	Total
**Humid**	Bizerte	Esseria	[39] (19)	[50] (30)	[29] (29)	[118] (78)
		Joumine				
		Meden				
		Sajnene				
	Jendouba	Bhira	[0] (0)	[14] (14)	[0] (0)	[14] (14)
	Beja	Amdoun	[56] (56)	[53] (43)	[30] (30)	[139] (129)
		Cap negro				
		El jouza				
		Nefza				
**Sub-Humid**	Bizerte	Utique	[22] (22)	[10] (10)	[0] (0)	[32] (32)
	Nabeul	Oued abid	[77] (27)	[38] (28)	[23] (22)	[138] (77)
		Takelsa				
	Kef	Mellegue	[0] (0)	[48] (28)	[0] (0)	[48] (28)
		Touiref				
**Semi-arid**	Ariana	Hessiene	[60] (60)	[28] (20)	[20] (20)	[108] (100)
		Kalaatlandlos				
		Sidi thabet				
	Nabeul	Solimaan	[20] (20)	[20] (10)	[20] (20)	[60] (50)
		Somaa				
		Diar ben Selem				
	Tunis	Hrairia	[26] (26)	[30] (18)	[30] (30)	[86] (74)
		Borjchakir				
		Sidi bechir				
	Zaghouan	Jouf 1	[63] (45)	[34] (34)	[14] (14)	[111] (93)
		Jouf 2				
**Arid**	Kairouan	Kairouan	[25] (24)	[0] (0)	[0] (0)	[25] (24)
**Total**	9	26	[388] (299)	[325] (235)	[166] (165)	[879] (699)

**Table 2:** Seroprevalence of RVFV and CCHFV in cattle, sheep and goats in the four bioclimatic zones.

Bioclimatic Zones	Localities	Antibodydetection of RVFV				Antibodydetection of CCHFV			
		Cattle Positive/Tested (%)	Sheep Positive/Tested (%)	Goats Positive/Tested (%)	Total % [CI95%]	Cattle Positive/Tested (%)	Sheep Positive/Tested (%)	Goats Positive/Tested (%)	Total % [CI95%]
**Humid**	Esseria Joumine Meden Sajnene Bhira Amdoun Cap negro El jouza Nefza	2/75 (2.7)	0/87 (0)	0/59 (0)	2/221 (0.9) [0–2.15]	0/95 (0)	0/117 (0)	0/59 (0)	0/271 (0) [0–1.1]
**Sub-humid**	Utique Oued abid Takelsa Mellegue Touiref	1/49 (2)	4/66 (6.1)	0/22 (0)	5/137 (3.6) [0.51–6.79]	18/99 (18.2)	20/96 (20.8)	13/23 (56.5)	51/218 (23.4) [17.77–29.01]
**Semi-arid**	Hessiene Kalaat landlos Sidi thabet Solimaan Somaa Diar ben Selem Hrairia Borjchakir Sidi bechir Jouf 1 Jouf 2	7/151 (4.6)	2/82 (2.4)	0/84 (0)	9/317 (2.8) [1.01–4.66]	24/169 (14.2)	0/112 (0)	0/84 (0)	24/365 (6.5) [4.03–9.11]
**Arid**	Kairouan	0/24 (0)	0/0 (0)	0/0 (0)	0/24 (0) [0–12.5]	1/25 (4)	0/0 (0)	0/0 (0)	1/25 (4) [0–11.68]
**Total**		10/299 (3.3)	6/235 (2.6)	0/165 (0)	16/699 (2.3) [1.18–3.39]	43/388 (11.1)	20/325 (6.2)	13/166 (7.8)	76/879 (8.6) [6.78–10.50]

**Table 3:** Univariate analysis of the association between risk factors and CCHFV seropositivity in cattle, sheep and goats in Tunisia.

Risk Factors	Cattle				Sheep				Goats			
	Categories	No. of Seropositive/No. of Tested Cattle (%)	[CI95%]	*p*-Value (OR) [CI95%]	Categories	No. of Seropositive/No. of Tested Cattle (%)	[CI95%]	*p*-Value (OR) [CI95%]	Categories	No. of Seropositive/No. of Tested Cattle (%)	[CI95%]	*p*-Value (OR) [CI95%]
**Breed**	Friesian Holtein local Local cross Pie noire Schwytz	3/27 (11.1) 8/120 (6.7) 22/102 (21.6) 4/74 (5.4) 1/20 (5) 5/45 (11.1)	[0–22.96] [2.2–11.13] [13.58–29.55] [0.25–10.55] [0–14.55] [1.92–20.29]	0.005	Barbarine Black Thibar Local cross QFO	6/252(2.4) 0/32 (0) 0/16 (0) 14/25 (56)	[0.49–4.26] [0–9.37] [0–18.75] [36.54–75.45]	<0.001	Damascus Local cross Maltese	0/55 (0) 13/108 (12) 0/3 (0)	[0–5.45] [5.9–18.17] [0–100]	0.023
**Age**	Adult Young	39/286 (13.6) 4/102 (3.9)	[9.65–17.61] [0.15–7.68]	0.007 (0.259) [0.09–0.743]	Adult Young	19/294 (6.5) 1/31 (3.2)	[3.65–9.27] [0–9.44]	0.476 (0.482) [0.062–3.732]	Adult Young	11/132 (8.3) 2/34 (5.9)	[3.61–13.04] [0–13.79]	0.635 (0.688) [0.145–3.259]
**Sex**	Female Male	41/338 (12.1) 2/50 (4)	[8.65–15.61] [0–9.43]	0.087 (0.302) [0.071–1.289]	Female Male	19/304 (6.3) 1/21 (4.8)	[3.52–8.97] [0–13.87]	0.784 (0.750) [0.095–5.892]	Female Male	10/150 (6.7) 3/16 (18.8)	[2.67–10.65] [0–37.87]	0.087 (3.231) [0.789–13.231]
**Season**	Autumn Spring Summer Winter	42/276 (15.2) 0/12 (0) 1/64 (1.6) 0/36 (0)	[10.98–19.45] [0–25] [0–4.6] [0–8.33]	0.001	Autumn Spring Summer Winter	18/172(10.5) 0/8 (0) 2/125 (1.6) 0/20 (0)	[5.89–15.04] [0–37.5] [0–3.8] [0–15]	0.008	Autumn Spring Summer Winter	13/133 (9.8) 0/9 (0) 0/14 (0) 0/10 (0)	[4.72–14.82] [0–33.33] [0–21.42] [0–30]	0.321
**Bioclimatic zones**	Humid Semi-arid Sub-humid Arid	0/95 (0) 24/169 (14.2) 18/99 (18.2) 1/25 (4)	[0–3.15] [8.93–19.46] 10.58–25.78] [0–11.68]	<0.001	Humid Semi-arid Sub-humid	0/117 (0) 0/112 (0) 20/96 (20.8)	[0–2.56] [0–2.67] [12.7–28.95]	<0.001	Humid Semi-arid Sub-humid	0/59 (0) 0/84 (0) 13/23(56.5)	[0–5.08] [0–3.57] [36.26–76.78]	<0.001
**Tick infestation**	No Yes	3/140 (2.1) 18/65 (27.7)	[0–4.54] [16.81–38.57]	<0.001 (17.489) [4.929–62.051]	No Yes	6/182 (3.3) 0/33 (0)	[0.70–5.89] [0–9.09]	0.29 (0.976) [0.941–0.993]	No Yes	6/96 (6.3) 7/10(70)	[1.40–11.09] [41.59–98.40]	<0.001 (35) [7.173–170.773]
**Type of breeding**	Modern Traditional	16/154 (10.4) 27/234 (11.5)	[5.57–15.2] [7.44–15.63]	0.724 (1.125) [0.584–2.165]	Traditional	ND	ND	ND	Traditional	ND	ND	ND

OR: odds ratio; ND: not determined.

**Table 4:** Univariate analysis of the association between risk factors and RVFV seropositivity in cattle and sheep in Tunisia.

Risk Factors	Cattle				Sheep			
	Categories	No. of Seropositive/No. of Tested Cattle (%)	[CI95%]	*p*-Value (OR) [CI95%]	Categories	No. of Seropositive/No. of Tested Sheep (%)	[CI95%]	*p*-Value (OR) [CI95%]
**Breed**	Friesian Holtein Local Local cross Pie noire Schwytz	0/20 (0) 5/114 (4.4) 5/81 (6.2) 0/49 (0) 0/19 (0) 0/16 (0)	[0–15] [0.62–8.14] [0.93–11.41] [0–6.12] [0–15.78] [0–18.75]	0.308	Barbarine Black Thibar Local cross QFO	4/182 (2.2) 2/21 (9.5) 0/14 (0) 0/18 (0)	[0.06–4.32] [0–22.07] [0–21.42] [0–16.66]	0.169
**Age**	Adult Young	7/224 (3.1) 3/75 (4)	[0.84–5.40] [0–8.43]	0.715 (1.292) [0.325–5.127]	Adult Young	2/213 (0.9) 4/22 (18.2)	[0–2.23] [2.06–34.29]	<0.001 (23.444) [4.016–136.855]
**Sex**	Female Male	8/265 (3) 2/34 (5.9)	[0.95–5.07] [0–13.79]	0.382 (2.008) [0.408–9.870]	Female Male	5/225 (2.2) 1/10 (10)	[0.29–4.14] [0–28.59]	0.127 (4.889) [0.516–46.297]
**Season**	Autumn Spring Summer Winter	8/198 (4) 0/12 (0) 2/63 (3.2) 0/26 (0)	[1.29–6.78] [0–25] [0–7.5] [0–11.53]	0.656	Autumn Spring Summer Winter	4/120 (3.3) 0/8 (0) 0/105 (0) 2/2 (100)	[0.12–6.54] [0–37.5] [0–2.85] [0–100]	<0.001
**Bioclimatic zones**	Humid Semi-arid Sub-humid Arid	2/75 (2.7) 7/151 (4.6) 1/49 (2) 0/24 (0)	[0–6.31] [1.28–7.98] [0–5] [0–12.5]	0.578	Humid Semi-arid Sub-humid	0/87 (0) 2/82 (2.4) 4/66 (6.1)	[0–3.44] [0–5.77] [0.3–11.81]	0.062
**Type of breeding**	Modern Traditionel	4/108 (3.7) 6/191 (3.1)	[0.14–7.26] [0.66–5.61]	0.795 (0.843) [0.233–3.056]	Traditional	ND	ND	ND

OR: odds ratio; ND: not determined.

## References

[1] Abudurexiti A., Adkins S., Alioto D., Alkhovsky S.V., Avšič-Županc T., Ballinger M.J., Bente D.A., Beer M., Bergeron É., Blair C.D. (2019). Taxonomy of the order Bunyavirales: Update 2019. Arch. Virol..

[2] Spengler J.R., Estrada-Peña A., Garrison A.R., Schmaljohn C., Spiropoulou C.F., Bergeron É., Bente D.A. (2016). A chronological review of experimental infection studies of the role of wild animals and livestock in the maintenance and transmission of Crimean-Congo hemorrhagic fever virus. Antiviral Res..

[3] Zoonotic Disease: Emerging Public Health Threats in the Region. www.emro.who.int/fr/about-who/rc61/zoonotic-diseases.html.

[4] Ergönül O. (2006). Crimean-Congo haemorrhagic fever. Lancet Infect. Dis..

[5] Hawman D.W., Feldmann H. (2018). Recent advances in understanding Crimean-Congo hemorrhagic fever virus. F1000Res.

[6] Spengler J.R., Bergeron É., Rollin P.E. (2016). Seroepidemiological Studies of Crimean-Congo Hemorrhagic Fever Virus in Domestic and Wild Animals. PLoS Negl. Trop. Dis..

[7] Zeller H.G., Cornet J.P., Camicas J.L. (1994). Experimental transmission of Crimean-Congo hemorrhagic fever virus by west African wild ground-feeding birds to Hyalomma marginatum rufipes ticks. Am. J. Trop. Med. Hyg..

[8] Bente D.A., Forrester N.L., Watts D.M., McAuley A.J., Whitehouse C.A., Bray M. (2013). Crimean-Congo hemorrhagic fever: History, epidemiology, pathogenesis, clinical syndrome and genetic diversity. Antivir. Res..

[9] Hoogstraal H. (1979). The epidemiology of tick-borne Crimean-Congo hemorrhagic fever in Asia, Europe, and Africa. J. Med. Entomol..

[10] Spengler J.R., Bergeron É., Spiropoulou C.F. (2019). Crimean-Congo hemorrhagic fever and expansion from endemic regions. Curr. Opin. Virol..

[11] Estrada-Peña A., Ayllón N., de la Fuente J. (2012). Impact of climate trends on tick-borne pathogen transmission. Front. Physiol..

[12] Monsalve Arteaga L., Muñoz Bellido J.L., Vieira Lista M.C., Vicente Santiago M.B., Fernández Soto P., Bas I., Leralta N., de Ory Manchón F., Negredo A.I., Sánchez Seco M.P. (2020). Crimean-Congo haemorrhagic fever (CCHF) virus-specific antibody detection in blood donors, Castile-León, Spain, summer 2017 and 2018. Euro. Surveill..

[13] Palomar A.M., Portillo A., Santibáñez P., Mazuelas D., Arizaga J., Crespo A., Gutiérrez Ó., Cuadrado J.F., Oteo J.A. (2013). Crimean-Congo hemorrhagic fever virus in ticks from migratory birds, Morocco. Emerg. Infect. Dis..

[14] Kautman M., Tiar G., Papa A., Široký P. (2016). AP92-like Crimean-Congo Hemorrhagic Fever Virus in Hyalomma aegyptium Ticks, Algeria. Emerg. Infect. Dis..

[15] Morrill J.C., Soliman A.K., Imam I.Z., Botros B.A., Moussa M.I., Watts D.M. (1990). Serological evidence of Crimean-Congo haemorrhagic fever viral infection among camels imported into Egypt. J. Trop. Med. Hyg..

[16] Mohamed M., Said A.-R., Murad A., Graham R. (2008). A serological survey of Crimean-Congo haemorrhagic fever in animals in the Sharkia Governorate of Egypt. Vet. Ital..

[17] Wasfi F., Dowall S., Ghabbari T., Bosworth A., Chakroun M., Varghese A., Tiouiri H., Ben Jemaa M., Znazen A., Hewson R. (2016). Sero-epidemiological survey of Crimean-Congo hemorrhagic fever virus in Tunisia. Parasite.

[18] Bouaicha F., Eisenbarth A., Elati K., Schulz A., Ben Smida B., Bouajila M., Sassi L., Rekik M., Groschup M.H., Khamassi Khbou M. (2021). Epidemiological investigation of Crimean-Congo haemorrhagic fever virus infection among the one-humped camels (Camelus dromedarius) in southern Tunisia. Ticks Tick Borne Dis..

[19] Linthicum K.J., Britch S.C., Anyamba A. (2016). Rift Valley Fever: An Emerging Mosquito-Borne Disease. Annu. Rev. Entomol..

[20] Hartman A. (2017). Rift Valley Fever. Clin. Lab. Med..

[21] Arishi H.M., Aqeel A.Y., Al Hazmi M.M. (2006). Vertical transmission of fatal Rift Valley fever in a newborn. Ann. Trop. Paediatr..

[22] Adam I., Karsany M.S. (2008). Case report: Rift Valley Fever with vertical transmission in a pregnant Sudanese woman. J. Med. Virol..

[23] Lumley S., Horton D.L., Hernandez-Triana L.L.M., Johnson N., Fooks A.R., Hewson R. (2017). Rift Valley fever virus: Strategies for maintenance, survival and vertical transmission in mosquitoes. J. Gen. Virol..

[24] Tong C., Javelle E., Grard G., Dia A., Lacrosse C., Fourié T., Gravier P., Watier-Grillot S., Lancelot R., Letourneur F. (2019). Tracking Rift Valley fever: From Mali to Europe and other countries, 2016. Euro Surveill..

[25] Wilson M.L. (1994). Rift Valley fever virus ecology and the epidemiology of disease emergence. Ann. N. Y. Acad. Sci..

[26] Daubney R. (1931). Enzootic hepatitis or Rift Valley fever: An undescribed virus disease of sheep cattle and man from East Africa. J. Path. Bact..

[27] Van Velden D.J., Meyer J.D., Olivier J., Gear J.H., McIntosh B. (1977). Rift Valley fever affecting humans in South Africa: A clinicopathological study. S. Afr. Med. J..

[28] Gad A.M., Feinsod F.M., Allam I.H., Eisa M., Hassan A.N., Soliman B.A., el Said S., Saah A.J. (1986). A possible route for the introduction of Rift Valley fever virus into Egypt during 1977. J. Trop. Med. Hyg..

[29] Monath T.P. (2020). The Arboviruses: Epidemiology and Ecology.

[30] Ayari-Fakhfakh E., Ghram A., Bouattour A., Larbi I., Gribâa-Dridi L., Kwiatek O., Bouloy M., Libeau G., Albina E., Cêtre-Sossah C. (2011). First serological investigation of peste-des-petits-ruminants and Rift Valley fever in Tunisia. Vet. J..

[31] Selmi R., Mamlouk A., Ben Said M., Ben Yahia H., Abdelaali H., Ben Chehida F., Daaloul-Jedidi M., Gritli A., Messadi L. (2020). First serological evidence of the Rift Valley fever Phlebovirus in Tunisian camels. Acta Trop..

[32] Bosworth A., Ghabbari T., Dowall S., Varghese A., Fares W., Hewson R., Zhioua E., Chakroun M., Tiouiri H., Ben Jemaa M. (2016). Serologic evidence of exposure to Rift Valley fever virus detected in Tunisia. New Microbes. New Infect..

[33] Moutailler S., Krida G., Schaffner F., Vazeille M., Failloux A.-B. (2008). Potential vectors of Rift Valley fever virus in the Mediterranean region. Vector Borne Zoonotic Dis..

[34] Krida G., Diancourt L., Bouattour A., Rhim A., Chermiti B., Failloux A.-B. (2011). Estimation du risque d’introduction du virus de la fièvre de la vallée du Rift en Tunisie par le moustique Culex pipiens. Bull. Soc. Pathol. Exot..

[35] Amraoui F., Krida G., Bouattour A., Rhim A., Daaboub J., Harrat Z., Boubidi S.-C., Tijane M., Sarih M., Failloux A.-B. (2012). Culex pipiens, an experimental efficient vector of West Nile and Rift Valley fever viruses in the Maghreb region. PLoS ONE.

[36] Bouattour A., Darghouth M.A., Daoud A. (1999). Distribution and ecology of ticks (Acari: Ixodidae) infesting livestock in Tunisia: An overview of eighth years field collections. Parassitologia.

[37] Beji M., Rhim A., Roiz D., Bouattour A. (2017). Ecophysiological characterization and molecular differentiation of Culex pipiens forms (Diptera: Culicidae) in Tunisia. Parasit Vectors.

[38] Okely M., Anan R., Gad-Allah S., Samy A.M. (2020). Mapping the environmental suitability of etiological agent and tick vectors of Crimean-Congo hemorrhagic fever. Acta Trop..

[39] Deyde V.M., Khristova M.L., Rollin P.E., Ksiazek T.G., Nichol S.T. (2006). Crimean-Congo hemorrhagic fever virus genomics and global diversity. J. Virol..

[40] Ozkaya E., Dincer E., Carhan A., Uyar Y., Ertek M., Whitehouse C.A., Ozkul A. (2010). Molecular epidemiology of Crimean-Congo hemorrhagic fever virus in Turkey: Occurrence of local topotype. Virus Res..

[41] Papa A., Sidira P., Larichev V., Gavrilova L., Kuzmina K., Mousavi-Jazi M., Mirazimi A., Ströher U., Nichol S. (2014). Crimean-Congo Hemorrhagic Fever Virus, Greece. Emerg. Infect. Dis..

[42] Elevli M., Ozkul A.A., Civilibal M., Midilli K., Gargili A., Duru N.S. (2010). A newly identified Crimean-Congo hemorrhagic fever virus strain in Turkey. Int. J. Infect. Dis..

[43] Sherifi K., Cadar D., Muji S., Robaj A., Ahmeti S., Jakupi X., Emmerich P., Krüger A. (2014). Crimean-Congo hemorrhagic fever virus clades V and VI (Europe 1 and 2) in ticks in Kosovo, 2012. PLoS Negl. Trop. Dis..

[44] García Rada A. (2016). First outbreak of Crimean-Congo haemorrhagic fever in western Europe kills one man in Spain. BMJ.

[45] Negredo A., de la Calle-Prieto F., Palencia-Herrejón E., Mora-Rillo M., Astray-Mochales J., Sánchez-Seco M.P., Bermejo Lopez E., Menárguez J., Fernández-Cruz A., Sánchez-Artola B. (2017). Autochthonous Crimean-Congo Hemorrhagic Fever in Spain. N. Engl. J. Med..

[46] Camicas J.-L., Wilson M.L., Cornet J.-P., Digoutte J.-P., Calvo M.-A., Adam F., Gonzalez J.-P., Calisher C.H. (1991). Ecology of Ticks as Potential Vectors of Crimean-Congo Hemorrhagic Fever Virus in Senegal: Epidemiological Implications. Hemorrhagic Fever with Renal Syndrome, Tick- and Mosquito-Borne Viruses.

[47] Ibrahim A.M., Adam I.A., Osman B.T., Aradaib I.E. (2015). Epidemiological survey of Crimean Congo hemorrhagic fever virus in cattle in East Darfur State, Sudan. Ticks Tick Borne Dis..

[48] Schuster I., Chaintoutis S.C., Dovas C.I., Groschup M.H., Mertens M. (2017). Detection of Crimean-Congo hemorrhagic fever virus-specific IgG antibodies in ruminants residing in Central and Western Macedonia, Greece. Ticks Tick Borne Dis..

[49] Grech-Angelini S., Lancelot R., Ferraris O., Peyrefitte C.N., Vachiery N., Pédarrieu A., Peyraud A., Rodrigues V., Bastron D., Libeau G. (2020). Crimean-Congo Hemorrhagic Fever Virus Antibodies among Livestock on Corsica, France, 2014–2016. Emerg. Infect. Dis..

[50] Schuster I., Mertens M., Mrenoshki S., Staubach C., Mertens C., Brüning F., Wernike K., Hechinger S., Berxholi K., Mitrov D. (2016). Sheep and goats as indicator animals for the circulation of CCHFV in the environment. Exp. Appl. Acarol..

[51] Estrada-Peña A., de la Fuente J. (2014). The ecology of ticks and epidemiology of tick-borne viral diseases. Antiviral Res..

[52] Ostfeld R.S., Brunner J.L. (2015). Climate change and Ixodes tick-borne diseases of humans. Philos. Trans. R. Soc. Lond B Biol. Sci..

[53] Shepherd A.J., Swanepoel R., Cornel A.J., Mathee O. (1989). Experimental studies on the replication and transmission of Crimean-Congo hemorrhagic fever virus in some African tick species. Am. J. Trop. Med. Hyg..

[54] Bouattour A., Darghouth M.A., Ben Miled L. (1996). Cattle infestation by Hyalomma ticks and prevalence of Theileria in H. detritum species in Tunisia. Vet. Parasitol..

[55] Sidira P., Maltezou H.C., Haidich A.-B., Papa A. (2012). Seroepidemiological study of Crimean-Congo haemorrhagic fever in Greece, 2009–2010. Clin. Microbiol. Infect..

[56] Bakir M., Ugurlu M., Dokuzoguz B., Bodur H., Tasyaran M.A., Vahaboglu H., Turkish Cchf Study Group (2005). Crimean-Congo haemorrhagic fever outbreak in Middle Anatolia: A multicentre study of clinical features and outcome measures. J. Med. Microbiol..

[57] Albayrak H., Ozan E., Kurt M. (2012). Serosurvey and molecular detection of Crimean-Congo hemorrhagic fever virus (CCHFV) in northern Turkey. Trop. Anim. Health Prod..

[58] Papa A., Velo E., Papadimitriou E., Cahani G., Kota M., Bino S. (2009). Ecology of the Crimean-Congo hemorrhagic fever endemic area in Albania. Vector Borne Zoonotic Dis..

[59] Alavi-Naini R., Moghtaderi A., Koohpayeh H.-R., Sharifi-Mood B., Naderi M., Metanat M., Izadi M. (2006). Crimean-Congo hemorrhagic fever in Southeast of Iran. J. Infect..

[60] Sherifi K., Rexhepi A., Berxholi K., Mehmedi B., Gecaj R.M., Hoxha Z., Joachim A., Duscher G.G. (2018). Crimean-Congo Hemorrhagic Fever Virus and Borrelia burgdorferi sensu lato in Ticks from Kosovo and Albania. Front. Vet. Sci.

[61] Akuffo R., Brandful J.A.M., Zayed A., Adjei A., Watany N., Fahmy N.T., Hughes R., Doman B., Voegborlo S.V., Aziati D. (2016). Crimean-Congo hemorrhagic fever virus in livestock ticks and animal handler seroprevalence at an abattoir in Ghana. BMC Infect. Dis..

[62] Teshome T., Deneke Y., Ibrahim N. (2016). Prevalence and Species Composition of Ticks Infesting Cattle in and around Bishoftu Town. Oromia Reg. Ethiop. Glob. Vet..

[63] Adam I.A., Mahmoud M.A.M., Aradaib I.E. (2013). A seroepidemiological survey of Crimean Congo hemorrhagic fever among cattle in North Kordufan State, Sudan. Virol. J..

[64] Elati K., Hamdi D., Jdidi M., Rekik M., Gharbi M. (2018). Differences in tick infestation of Tunisian sheep breeds. Vet. Parasitol. Reg. Stud. Rep..

[65] Ahmadkhani M., Alesheikh A.A., Khakifirouz S., Salehi-Vaziri M. (2018). Space-time epidemiology of Crimean-Congo hemorrhagic fever (CCHF) in Iran. Ticks Tick Borne Dis..

[66] Choubdar N., Oshaghi M.A., Rafinejad J., Pourmand M.R., Maleki-Ravasan N., Salehi-Vaziri M., Telmadarraiy Z., Karimian F., Koosha M., Rahimi-Foroushani A. (2019). Effect of Meteorological Factors on Hyalomma Species Composition and Their Host Preference, Seasonal Prevalence and Infection Status to Crimean-Congo Haemorrhagic Fever in Iran. J. Arthropod. Borne Dis..

[67] Atif F.A., Khan M.S., Iqbal H.J., Ali Z., Ullah S. (2012). Prevalence of cattle tick infestation in three districts of the Punjab, Pakistan. Pak. J. Sci..

[68] Lotfollahzadeh S., Nikbakht Boroujeni G.R., Mokhber Dezfouli M.R., Bokaei S. (2011). A serosurvey of Crimean-Congo haemorrhagic fever virus in dairy cattle in Iran. Zoonoses Public Health.

[69] Papa A., Sidira P., Kallia S., Ntouska M., Zotos N., Doumbali E., Maltezou H.C., Demiris N., Tsatsaris A. (2013). Factors associated with IgG positivity to Crimean-Congo hemorrhagic fever virus in the area with the highest seroprevalence in Greece. Ticks Tick Borne Dis..

[70] El-Harrak M., Martín-Folgar R., Llorente F., Fernández-Pacheco P., Brun A., Figuerola J., Jiménez-Clavero M.A. (2011). Rift Valley and West Nile virus antibodies in camels, North Africa. Emerg. Infect. Dis..

[71] Di Nardo A., Rossi D., Saleh S.M.L., Lejlifa S.M., Hamdi S.J., Di Gennaro A., Savini G., Thrusfield M.V. (2014). Evidence of Rift Valley fever seroprevalence in the Sahrawi semi-nomadic pastoralist system, Western Sahara. BMC Vet. Res..

[72] Centers for Disease Control and Prevention (CDC) (2000). Outbreak of Rift Valley fever—Saudi Arabia, August-October, 2000. Morb. Mortal. Wkly. Rep..

[73] Asebe G., Mamo G., Michlmayr D., Abegaz W.E., Endale A., Medhin G., Larrick J.W., Legesse M. (2020). Seroprevalence of Rift Valley Fever and West Nile Fever in Cattle in Gambella Region, South West Ethiopia. Vet. Med..

[74] Clark M.H.A., Warimwe G.M., Di Nardo A., Lyons N.A., Gubbins S. (2018). Systematic literature review of Rift Valley fever virus seroprevalence in livestock, wildlife and humans in Africa from 1968 to 2016. PLoS Negl. Trop. Dis..

[75] Arsevska E., Hellal J., Mejri S., Hammami S., Marianneau P., Calavas D., Hénaux V. (2016). Identifying Areas Suitable for the Occurrence of Rift Valley Fever in North Africa: Implications for Surveillance. Transbound. Emerg. Dis..

[76] Redding D.W., Tiedt S., Lo Iacono G., Bett B., Jones K.E. (2017). Spatial, seasonal and climatic predictive models of Rift Valley fever disease across Africa. Philos. Trans. R. Soc. Lond B Biol. Sci..

[77] Amara Korba R., Alayat M.S., Bouiba L., Boudrissa A., Bouslama Z., Boukraa S., Francis F., Failloux A.-B., Boubidi S.C. (2016). Ecological differentiation of members of the Culex pipiens complex, potential vectors of West Nile virus and Rift Valley fever virus in Algeria. Parasit Vectors.

[78] Brustolin M., Talavera S., Nuñez A., Santamaría C., Rivas R., Pujol N., Valle M., Verdún M., Brun A., Pagès N. (2017). Rift Valley fever virus and European mosquitoes: Vector competence of Culex pipiens and Stegomyia albopicta (=Aedes albopictus). Med. Vet. Entomol..

[79] Bouattour A., Khrouf F., Rhim A., M’ghirbi Y. (2019). First Detection of the Asian Tiger Mosquito, Aedes (Stegomyia) albopictus (Diptera: Culicidae), in Tunisia. J. Med. Entomol..

[80] Soumare B., Tempia S., Cagnolati V., Mohamoud A., Van Huylenbroeck G., Berkvens D. (2007). Screening for Rift Valley fever infection in northern Somalia: A GIS based survey method to overcome the lack of sampling frame. Vet. Microbiol..

[81] Blomström A.-L., Scharin I., Stenberg H., Figueiredo J., Nhambirre O., Abilio A., Berg M., Fafetine J. (2016). Seroprevalence of Rift Valley fever virus in sheep and goats in Zambézia, Mozambique. Infect. Ecol. Epidemiol..

[82] Nyakarahuka L., de St Maurice A., Purpura L., Ervin E., Balinandi S., Tumusiime A., Kyondo J., Mulei S., Tusiime P., Lutwama J. (2018). Prevalence and risk factors of Rift Valley fever in humans and animals from Kabale district in Southwestern Uganda, 2016. PLoS Negl. Trop. Dis..

[83] Ngoshe Y.B., Avenant A., Rostal M.K., Karesh W.B., Paweska J.T., Bagge W., Jansen van Vuren P., Kemp A., Cordel C., Msimang V. (2020). Patterns of Rift Valley fever virus seropositivity in domestic ruminants in central South Africa four years after a large outbreak. Sci. Rep..

[84] Pepin M., Bouloy M., Bird B.H., Kemp A., Paweska J. (2010). Rift Valley fever virus (Bunyaviridae: Phlebovirus): An update on pathogenesis, molecular epidemiology, vectors, diagnostics and prevention. Vet. Res..

[85] Paweska J.T., Burt F.J., Anthony F., Smith S.J., Grobbelaar A.A., Croft J.E., Ksiazek T.G., Swanepoel R. (2003). IgG-sandwich and IgM-capture enzyme-linked immunosorbent assay for the detection of antibody to Rift Valley fever virus in domestic ruminants. J. Virol. Methods.

[86] Morvan J., Rollin P.E., Laventure S., Roux J. (1992). Duration of immunoglobulin M antibodies against Rift Valley fever virus in cattle after natural infection. Trans. R. Soc. Trop. Med. Hyg..

[87] Jäckel S., Eiden M., El Mamy B.O., Isselmou K., Vina-Rodriguez A., Doumbia B., Groschup M.H. (2013). Molecular and serological studies on the Rift Valley fever outbreak in Mauritania in 2010. Transbound. Emerg. Dis..

[88] Mertens M., Vatansever Z., Mrenoshki S., Krstevski K., Stefanovska J., Djadjovski I., Cvetkovikj I., Farkas R., Schuster I., Donnet F. (2015). Circulation of Crimean-Congo Hemorrhagic Fever Virus in the former Yugoslav Republic of Macedonia revealed by screening of cattle sera using a novel enzyme-linked immunosorbent assay. PLoS Negl. Trop. Dis..

[89] Sas M.A., Comtet L., Donnet F., Mertens M., Vatansever Z., Tordo N., Pourquier P., Groschup M.H. (2018). A novel double-antigen sandwich ELISA for the species-independent detection of Crimean-Congo hemorrhagic fever virus-specific antibodies. Antivir. Res..

[90] Kortekaas J., Kant J., Vloet R., Cêtre-Sossah C., Marianneau P., Lacote S., Banyard A.C., Jeffries C., Eiden M., Groschup M. (2013). European ring trial to evaluate ELISAs for the diagnosis of infection with Rift Valley fever virus. J. Virol. Methods.

